# A subset of conserved mammalian long non-coding RNAs are fossils of ancestral protein-coding genes

**DOI:** 10.1186/s13059-017-1293-0

**Published:** 2017-08-30

**Authors:** Hadas Hezroni, Rotem Ben-Tov Perry, Zohar Meir, Gali Housman, Yoav Lubelsky, Igor Ulitsky

**Affiliations:** Department of Biological Regulation, Weizmann Institute of Science, 234 Herzl St., Rehovot, 76100 Israel

**Keywords:** Long noncoding RNAs, Evolution, Pseudogenes, Translational regulation, uORFs, X inactivation

## Abstract

**Background:**

Only a small portion of human long non-coding RNAs (lncRNAs) appear to be conserved outside of mammals, but the events underlying the birth of new lncRNAs in mammals remain largely unknown. One potential source is remnants of protein-coding genes that transitioned into lncRNAs.

**Results:**

We systematically compare lncRNA and protein-coding loci across vertebrates, and estimate that up to 5% of conserved mammalian lncRNAs are derived from lost protein-coding genes. These lncRNAs have specific characteristics, such as broader expression domains, that set them apart from other lncRNAs. Fourteen lncRNAs have sequence similarity with the loci of the contemporary homologs of the lost protein-coding genes. We propose that selection acting on enhancer sequences is mostly responsible for retention of these regions. As an example of an RNA element from a protein-coding ancestor that was retained in the lncRNA, we describe in detail a short translated ORF in the JPX lncRNA that was derived from an upstream ORF in a protein-coding gene and retains some of its functionality.

**Conclusions:**

We estimate that ~ 55 annotated conserved human lncRNAs are derived from parts of ancestral protein-coding genes, and loss of coding potential is thus a non-negligible source of new lncRNAs. Some lncRNAs inherited regulatory elements influencing transcription and translation from their protein-coding ancestors and those elements can influence the expression breadth and functionality of these lncRNAs.

**Electronic supplementary material:**

The online version of this article (doi:10.1186/s13059-017-1293-0) contains supplementary material, which is available to authorized users.

## Background

Genomic studies have revealed that vertebrate genomes encode thousands of genes that give rise to transcripts that closely resemble mRNAs on the molecular level, yet do not appear to encode any functional peptides. Collectively, these are referred to as long noncoding RNAs (lncRNAs). The fraction of lncRNAs that have any biological function is currently unclear, and those that are functional appear to act through diverse mechanisms in both the nucleus and the cytoplasm [1]. With the growing appreciation of the importance of some lncRNAs in various biological pathways, there is an increasing interest in understanding their evolution and in using comparative genomics to study their functional determinants [2].

We recently described a pipeline for identification of lncRNAs from RNA-seq data (PLAR) and applied it to data from various embryonic and adult tissues in 17 vertebrates [3]. Most lncRNAs in each species did not share any detectable similarity with lncRNAs in other species, suggesting rapid turnover of lncRNA repertoires, as also reported by others [4, 5]. Against this backdrop of high turnover, numerous lncRNAs are conserved between different vertebrates. Specifically, of the > 10,000 currently annotated human lncRNAs, ~ 100 have homologs in fish, ~ 300 in non-mammalian vertebrates, and over a thousand have sequence-similar counterparts in other mammals [3]. Many of the lncRNAs that are conserved only in mammals, such as XIST, HOTAIR, and NORAD, have established functions [6–9]. When did these loci start to produce lncRNAs and what was the nature of their DNA at that time? One possibility is that these lncRNAs are conserved outside of mammals, but the sequence similarity is so low that it is no longer detectable in contemporary species. The number of positionally conserved pairs of mammalian and non-mammalian lncRNAs indeed exceeds expectation [3, 10, 11], and the difference between the observed and the expected numbers of syntenic pairs between mammals and other vertebrates is larger than the number of pairs with sequence similarity [3]. However, this difference is small compared to the number of lncRNAs that do not have traceable homologs outside mammals, and so it is likely that many lncRNAs observed across mammals are mammalian innovations.

So far, events underlying the origin of new lncRNAs remain largely unknown [2]. Significant sequence similarity among lncRNAs in the same species is rare [3], and therefore it is unlikely that many lncRNAs evolved by gene duplication, the leading mechanism of diversification in proteins [12]. Two other possible sources of new lncRNAs are parts of protein-coding genes that lost their coding potential and untranscribed noncoding DNA that gained elements promoting production of stable transcripts, perhaps via adoption of sequences from transposable elements [1, 13]. We are focusing here on the first of these routes—fragments of protein-coding genes that lost coding capacity but retained some of the transcriptional control program, thus morphing from a protein-coding gene into a noncoding RNA. If the contemporary gene sequence resembles the coding sequence of the ancestor, the gene is likely to be annotated as an “unprocessed pseudogene” [14], and if there are no significant traces of the peptide sequence, as a lncRNA, and so those scenarios correspond to two regions in a continuum of coding sequence erosion. Previous studies have looked in detail at the potential noncoding functions of annotated transcribed pseudogenes in rodent [15], primate [16], and Poaceae lineages [17], but it has been difficult to estimate how many mammalian lncRNAs have protein-coding ancestry due to erosion of sequence similarity at large evolutionary distances. Three of the lncRNAs in the eutherian X-inactivation center—XIST, JPX, and FTX—are the only currently known examples of lncRNA genes born through this mechanism and retained across mammals [18, 19]. Here, we systematically assess the extent to which conserved mammalian lncRNAs were derived from parts of protein-coding genes that lost their coding capacity before the rise of mammals, the potential impact of such origin on lncRNA biology, and specific mechanisms that may underlie conservation of protein-coding sequence within lncRNA loci for over 100 million years.

## Results

### Identification of protein-coding genes lost prior to the emergence of mammals

**Fig. 1:**
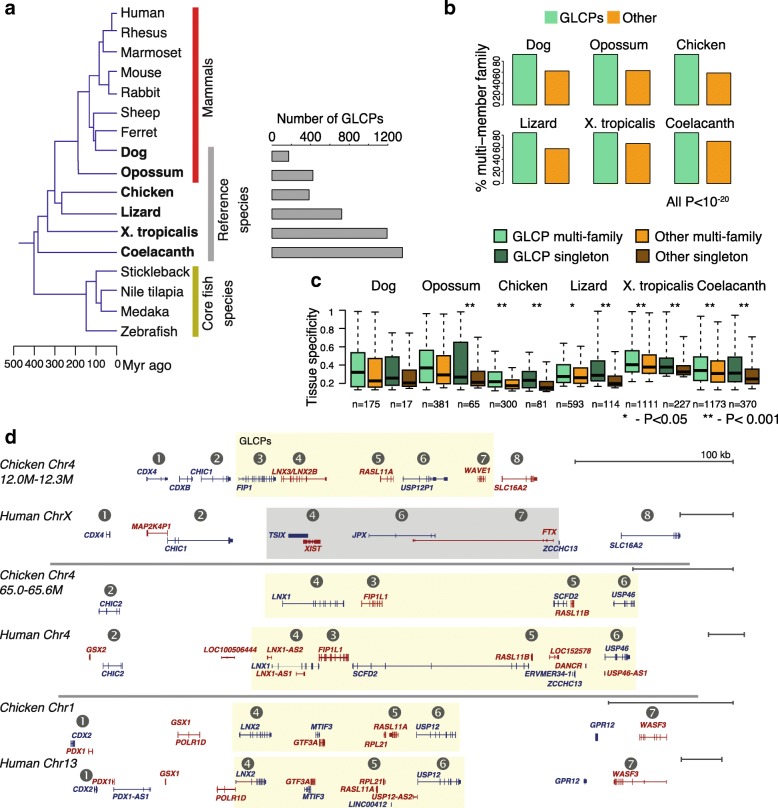
Protein-coding genes lost during vertebrate evolution. **a** Phylogeny of the species used for detection of “genes with lost coding potential” (*GLCPs*) and the numbers of GLCPs found in each of the reference species. **b** Fraction of GLCPs and other genes that belong to Ensembl protein families with more than one member. *P* values were computed using Pearson’s chi-squared test. **c** Tissue specificity indices [51] of the indicated groups of protein-coding genes in each of the reference species: (i) GLCPs belonging to Ensembl protein families with multiple members (*GLCP multi-family*); (ii) of other members of the same protein families (*Other multi-family*; the number of members from each family sampled to be as close as possible to the number of GLCPs in the family); (iii) of GLCPs that belong to families without additional members (*GLCP singleton*); and (iv) of other genes that belong to families without additional members (*Other singleton*). Numbers indicate the size of each group. All comparisons indicated by *asterisks* are significant at FDR < 0.05 (Benjamini–Hochberg method). **d** Genomic arrangements of genes in the three syntenic genomic clusters surrounding LNX genes in chicken and human. The shaded region highlights the genes in the X inactivation center (XIC), the GLCPs they were derived from, and their paralogs. Gene positions taken from the UCSC genome browser. For genes with multiple splice isoforms, a single representative transcript is shown. Gene model colors indicate the orientation of the gene. Circled numbers indicate assignment of genes to homology groups

In order to focus on high-confidence events of loss of protein-coding potential, we first systematically identified protein-coding genes that are missing from mammalian genomes yet are present in other vertebrates. We focused on species with relatively high-quality genome assemblies, including eight mammals (human, rhesus, marmoset, mouse, rabbit, dog, sheep, and ferret) and eight other vertebrates (chicken, anole lizard, *Xenopus tropicalis*, coelacanth, zebrafish, stickleback, tilapia, and medaka). To facilitate identification of protein-coding potential loss occurring at several time points during vertebrate evolution, we focused on six vertebrate species found at intermediate distances from human and mouse and used those as “reference species” in our analysis. Using Ensembl Compara [20], we identified groups of protein-coding genes found in opossum, chicken, anole lizard, *X. tropicalis*, and coelacanth, yet missing in eutherian mammals, and those found in dog and lost in primates and glires (Fig. 1a; Additional file 1: Figure S1; see “Methods”). Hundreds of genes met these criteria in each of the reference species (Fig. 1a; Additional file 2: Table S1) and we refer to these as “genes with lost coding potential” (GLCPs; note that coding potential is lost in mammals, and not in the reference species).

Coding potential loss in evolution can be facilitated by the presence of paralogous or related genes that can compensate for the consequences of the loss. Indeed, we found that GLCPs were much more likely than other genes to belong to an Ensembl protein family that had additional members in the reference species (Fig. 1b; *P* < 10^−15^ in all species). We also hypothesized that, among the members of these families, GLCPs may have carried out more specific, and hence more dispensable, roles at the time of their loss. Indeed, GLCPs are typically expressed at lower levels than the other members of their families and exhibit higher tissue specificity in the reference species (Fig. 1c; Additional file 1: Figure S2). The expression pattern at the time of protein-coding potential loss was not necessarily the same as that of the GLCP in the contemporary species, but the observed trend does suggest that family members with less global functional impact were more vulnerable to protein-coding potential loss and/or functional specialization during vertebrate evolution.

The ancestral gene cluster that gave rise to the X-inactivation center lncRNAs provides an illustrative example of how protein-coding potential loss may have been accommodated by presence of paralogous family members (Fig. 1d). The XIST, FTX, and JPX lncRNAs have been traced to their ancestral genes LNX3 (aka LNX2B), USPL, and WAVE1, respectively [18, 19], and these genes are indeed present in a conserved cluster throughout vertebrates, including in chicken (Fig. 1d), lizard, *Xenopus*, coelacanth, and spotted gar. Intriguingly, two additional genomic clusters containing paralogs of most of the genes in the extended LNX3/USPL/WAVE1 cluster are found throughout vertebrates (Fig. 1d), including the basal fish spotted gar.

### A significant number of GLCPs are syntenic with conserved mammalian lncRNAs

We hypothesized that, following loss of protein-coding potential, some GLCPs or parts of their loci retained or regained their ability to be transcribed and evolved into some of the mammalian lncRNAs. As lncRNA sequences evolve fast we relied on synteny as the main source of support for such events. Since coding potential loss presumably occurred early in mammalian evolution, we focused on 2740 and 1163 lncRNAs present in human or mouse genomes, respectively, and having significant sequence similarity with lncRNAs in other species (excluding human lncRNAs conserved in sequence only in primates; see “Methods”; Additional file 3: Table S2). The majority of 5′ and 3′ ends of transcript models of the conserved lncRNAs were experimentally supported by CAGE and 3P-seq data, respectively, and the conserved lncRNAs were generally better supported than other annotated lncRNAs (Additional file 1: Table S3).

**Fig. 2:**
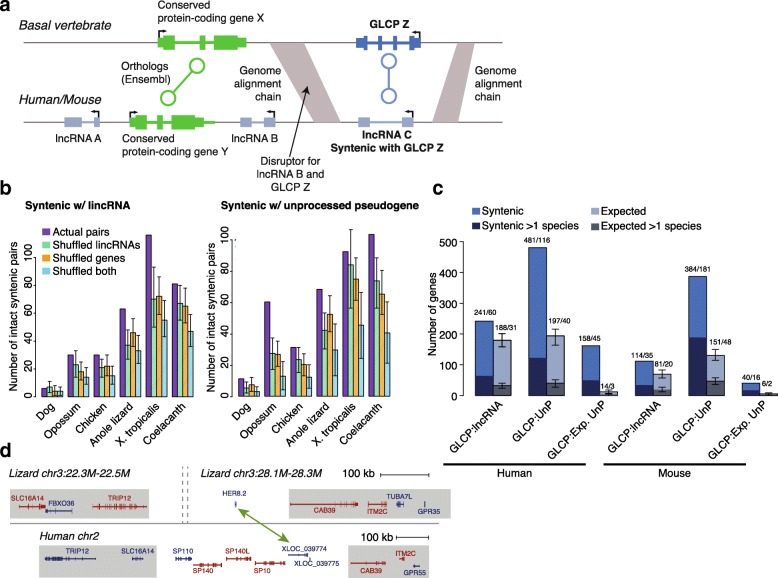
Using synteny to match GLCPs and conserved mammalian lncRNAs. **a** The methodology. **b** Numbers of positionally conserved GLCP–lncRNA pairs compared to the average numbers obtained when randomly placing lncRNAs (1000 iterations), selecting random groups of genes matched in number to the GLCPs (1000 iterations), or both (10,000 iterations). *Error bars* show empirical 90% confidence intervals. **c** Tallies of lncRNAs, unprocessed pseudogenes (*UnP*), and expressed unprocessed pseudogenes (*Exp. UnP*) syntenic with GLCPs in human and mouse. *>1 species* indicates that the lncRNA or the pseudogene were matched to a GLCP in more than one species. **d** Genomic organization of the human and lizard TRIP12/CAB39 loci

To identify lncRNAs potentially derived from GLCPs, we adapted an approach that we previously developed for identifying positionally conserved lncRNAs [3] to look for synteny between GLCPs in each of the reference species and lncRNAs (see “Methods”). The algorithm we employed works in two phases (Fig. 2a; Additional file 1: Figure S3a). First, when comparing a pair of species (a reference species vs. human or mouse), we looked for pairs of anchor protein-coding genes X (in a reference species) and Y (in human or mouse) such that (i) X and Y are orthologous to each other according to Ensembl; (ii) X is the flanking gene within a certain proximity (scaled by genome size) to a GLCP; (iii) Y is the flanking gene within a certain proximity to a conserved lncRNA; and (iv) the relative orientation of the GLCP to X and the lncRNA to Y is the same. In the second phase (also based on the syntenic lncRNA search methodology [3]), we used chains from the alignments of the genomes of two species to narrow down the syntenic regions and exclude inconsistent candidates. GLCP–lncRNA pairs for which we did not observe a “disrupting” chain (Additional file 1: Figure S3b; “Methods”) were considered positionally conserved. As expected, this second phase generally removed more spurious syntenic pairs between closer genomes (such as human–opossum) than between further ones (such as human–coelacanth) (Additional file 1: Figure S3c), where substantially smaller parts of the genomes were alignable.

We first verified that our approach is sufficiently sensitive. To do so, we tested if pairs of orthologous protein-coding genes were called as syntenic (after they were iteratively removed from the set of potential anchors). At least 69% of the pairs were properly recovered when comparing any of our six reference species with human and mouse (77% average recovery across all comparisons), and so the synteny-based analysis is powerful enough for recovery of most GLCP–lncRNA pairs.

The number of syntenic GLCP–lncRNA pairs exceeded that expected by chance by three different randomization tests in chicken, lizard, *X. tropicalis*, and coelacanth (empirical *P* < 0.05 in each; Fig. 2b; Additional file 4: Table S4; see Methods for a description of the randomizations), supporting the hypothesis that some conserved lncRNAs are GLCP-derived. When pooling together results across the reference species, we found 241 human and 114 mouse lncRNAs syntenic with a GLCP in at least one reference species (53 and 33 more than expected by chance; Fig. 2c). Out of these, 60 and 35 in human and mouse, respectively, were syntenic with GLCPs in at least two reference species. We thus estimate that ~ 2–3% of the lncRNAs annotated in human and mouse contain parts derived from ancestrally lost protein-coding genes. Following our focus on conserved lncRNAs, 152/241 (63%) of the human GLCP-derived lncRNAs had either a homolog in mouse (from [3]) or a corresponding mouse lncRNA derived from the same GLCP. Similarly, 74% of mouse GLCP-derived lncRNAs had a sequence-similar and/or corresponding human lncRNA. The same approach traced 491/384 annotated unprocessed pseudogenes in human/mouse to GLCPs, and 158/40 of these are expressed at appreciable levels (read per kilobase per million reads (RPKM) > 1; Fig. 2c; Additional file 4: Table S4). Derivation from GLCPs as a mechanism for birth of lncRNAs is thus more common than the three examples described in the literature so far, but GLCP-derived lncRNAs are still a small minority of the hundreds of lncRNAs conserved among mammals [3–5], suggesting that the vast majority of these evolved from noncoding DNA that gained transcription.

### Properties of GLCPs and putative GLCP-derived lncRNAs suggest possible mechanisms for loss of coding potential

It is difficult to estimate the events that led to loss of protein-coding potential as they presumably mostly occurred > 200 million years ago. One possible cause is a genomic rearrangement that disrupted the ancestral GLCP locus. Supporting the potential prevalence of such events, putative GLCP-derived lncRNAs overlapped breakpoint intervals—boundaries between stretches of consecutive orthologous genes when comparing human with other vertebrates [21]—more often than expected by chance (9.5% for lncRNAs syntenic with GLCPs vs. 5.4% for other lncRNAs, *P* = 0.0046, hypergeometric test). The number of putative GLCP-derived lncRNAs that overlapped the breakpoint intervals (23) was significant also when we randomly permuted the locations of the intervals in the human genome (eight overlaps expected by chance on average in 10,000 permutations, *P* < 10^−5^ for the number of overlaps and *P* = 7 × 10^−3^ for the enrichment of overlaps with GLCP-derived lncRNAs compared to other lncRNAs) and when we randomly permutated lncRNA locations (11 overlaps expected by chance in 10,000 permutations, *P* = 7 × 10^−4^). For example, the GLCP HER8.2 (ENSACAG00000002708 in lizard) is found in one such breakpoint region between the CAB39 and SLC16A14 genes, where two separate lncRNAs, XLOC_039774 and XLOC_039775, preserve synteny with respect to CAB39 (Fig. 2d).

**Fig. 3:**
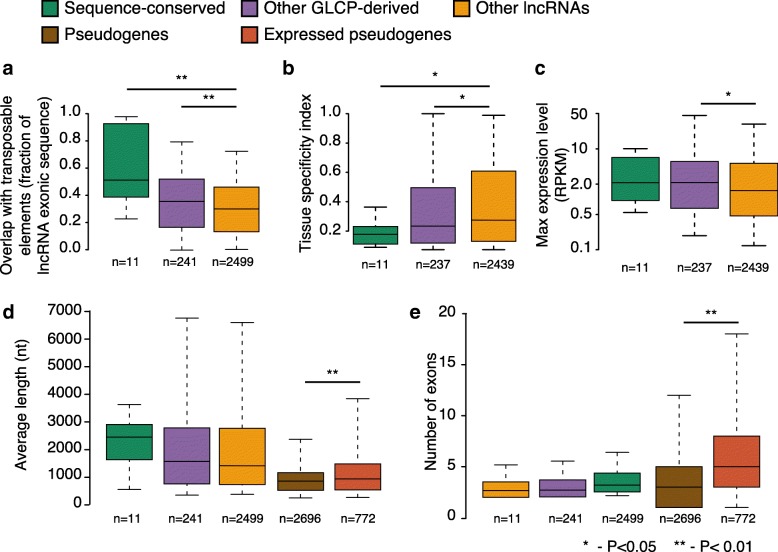
Characteristics of GLCP-derived lncRNAs and unprocessed pseudogenes. **a** Fraction of exonic sequence overlapping transposable elements identified by RepeatMasker. **b**, **c** Comparison of the tissue specificity index [51] (**b**) and mean expression (**c**) for GLCP-derived lncRNAs, divided into a set of lncRNAs with sequence similarity to GLCPs and a set of those without (*Other GLCP-derived*). **d**, **e** Comparison of exonic length (**d**) and number of exons (**e**), averaged across all isoforms for each gene, between the indicated groups of lncRNAs and unprocessed pseudogenes

Another potential cause of protein-coding potential loss is through exonization of transposable elements in the coding sequence that leads to ORF disruption. Indeed, a relatively large fraction of GLCP-derived lncRNA sequence overlapped transposable elements (Fig. 3a). Further, 34% of GLCP-derived lncRNAs had an isoform whose transcription start site overlapped a transposable element (not significantly different from other lncRNAs, *P* = 0.65), suggesting that in many cases the contemporary lncRNA promoter is not orthologous to the ancestral GLCP promoter but was rather adopted during or after the protein-coding potential loss. We note that this analysis might be affected by the partial completeness of the 5′ end annotations of the human and mouse lncRNAs (Additional file 1: Table S3).

### Sequence similarity between genes that lost coding potential and recycled lncRNAs is rare

We next used three different alignment methods to seek regions with significant sequence similarity between syntenic pairs. Two of the methods (BLASTN and SSEARCH) compare nucleic acid sequences, and another (TBLASTX) compares protein sequences translated in different frames from each transcript. We tested different thresholds for sequence similarity (Additional file 1: Figure S4) and selected a threshold of 10^−5^ for the BLAST comparisons (similar to the threshold we previously used for comparing lncRNA sequences [3]) and a conservative threshold of 10^−10^ when using the potentially more sensitive Smith–Waterman local alignment (SSEARCH implementation [22]). Under these thresholds, 11 GLCP–lncRNA pairs and 56 GLCP–pseudogene pairs in human had significant sequence similarity (20.7 and 19.7% of the number of positionally conserved pairs above background, FDR < 0.25). Best-scoring local alignment regions between the lncRNAs and GLCPs were quite short (158 bases on average for SSEARCH alignments). Three lncRNA–GLCP pairs without sequence similarity were supported by whole-genome alignments overlapping their loci (Additional file 4: Table S4). These results are consistent with the limited homology observed between XIST and LNX3 sequences from different species [18, 23, 24].

### Sequence and expression features of GLCP-derived genes

As further support for the connection between the GLCPs and the lncRNAs, in three of the species we found significant correlation between the expression levels of GLCPs and the lncRNAs putatively derived from them (Additional file 1: Figure S5). We next tested whether the potentially GLCP-derived lncRNAs differ from other conserved lncRNAs in their genomic features and expression domains. We note that our power in performing such comparisons is compromised by the relatively high FDR of our approach. Still, GLCP-derived lncRNAs were expressed more broadly and at higher expression levels than other lncRNAs (Fig. 3b, c), with further differences between sequence-conserved and other GLCP-derived lncRNAs. GLCP-derived lncRNAs were not significantly different from other lncRNAs in transcript length or number of exons (Fig. 3d, e). While pseudogenes were shorter than lncRNAs and had a similar number of exons, expressed pseudogenes (that can be considered a class of lncRNAs) were similar to annotated lncRNAs in their length but had significantly more exons (Fig. 3d, e).

### Sequence similarity in some cases might stem from overlap with enhancer elements

**Fig. 4:**
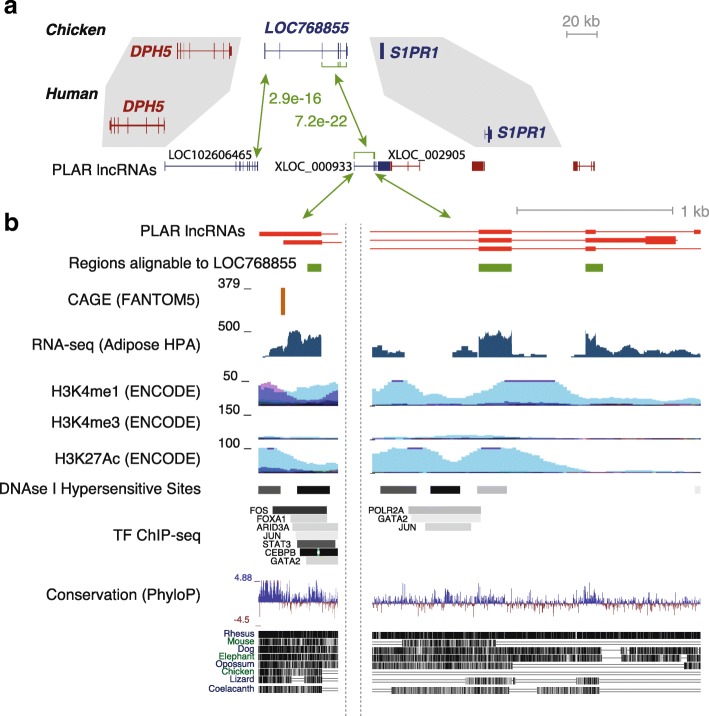
Part of the sequence conservation in GLCP–lncRNA pairs can be explained by overlap with enhancer elements. **a** Genomic organization of the DPH5/S1PR1 locus in human and chicken. *P* values are from SSEARCH comparisons of the sequences of the syntenic loci. Gene model colors indicate the orientation of the gene. **b** Detailed characterization of three of the exons of XLOC_000933: transcription start sites mapped using CAGE by the FANTOM5 consortium [54]; RNA-seq coverage in the adipose tissue from the Human Proteome Atlas [55]; chromatin modifications characteristic of enhancers (H3K4me1), promoters (H3K4me3), and active regulatory elements (H3K27ac); DNAse I hypersensitivity clusters; and transcription factor (*TF*) binding sites identified using ChIP-seq by the ENCODE project taken from the UCSC genome browser; base-wise sequence conservation and multiple alignment of different vertebrates taken from the UCSC genome browser

Some of the cases of sequence conservation between GLCPs and GLCP-derived lncRNAs could be related to overlap of GLCP loci with enhancer elements that create further sequence constraints. An illustrative example of such a GLCP is the chicken LOC768855 (ENSGALG00000020884), a six-exon protein-coding gene located between SP1R1 and DPH5 and assigned in Ensembl to the PROSTAGLANDIN F2 ALPHA SYNTHASE family (Fig. 4a). One-to-one orthologs of LOC768855 are found in 17 non-mammalian vertebrates in Ensembl Compara, but no homologs are found in any of 39 placental mammals. Two human lncRNAs are syntenic to LOC768855 and share significant sequence similarity with it: LOC102606465, a broadly expressed lncRNA transcribed from a promoter proximal to DPH5 in a divergent orientation; and XLOC_000933, a lncRNA we annotated using PLAR [3] that is predominantly expressed in adipose tissue. Some of the RNA-seq-reconstructed isoforms of LOC768855 in chicken begin from a promoter proximal to DPH5, and therefore the two human lncRNA together quite closely correspond to the ancestral protein-coding gene. Similar lncRNAs are found in dog and sheep, but in glires we found only a shorter transcript, which is divergent with DPH5 and does not share sequence similarity with LOC768855. Interestingly, most of the regions of sequence similarity between XLOC_000933 and LOC102606465 overlap regions of significant enhancer activity in human cells (demarcated by H3K4me1 and H3K27ac chromatin marks; Fig. 4b). It is unknown if the exons of LOC768855 also act as enhancers, so it is unclear if these enhancer elements predate the loss of LOC768855 in mammals. Sequence conservation between the GLCP and the lncRNA in this and potentially other cases may thus stem from selective pressures to preserve specific DNA elements necessary for enhancer activity. Notably, however, transcription of the two lncRNAs in this locus, as well as their specific splice sites, appear to be highly conserved in mammals, suggesting functional importance of the lncRNA itself. We conclude that it is likely that some of the cases of rather extensive sequence similarity between the GLCP and the GLCP-derived lncRNA stem from the importance of the sequences as DNA elements, such as enhancers or insulators.

### An upstream ORF in USPL is recycled as a regulatory element in the JPX lncRNA

**Fig. 5:**
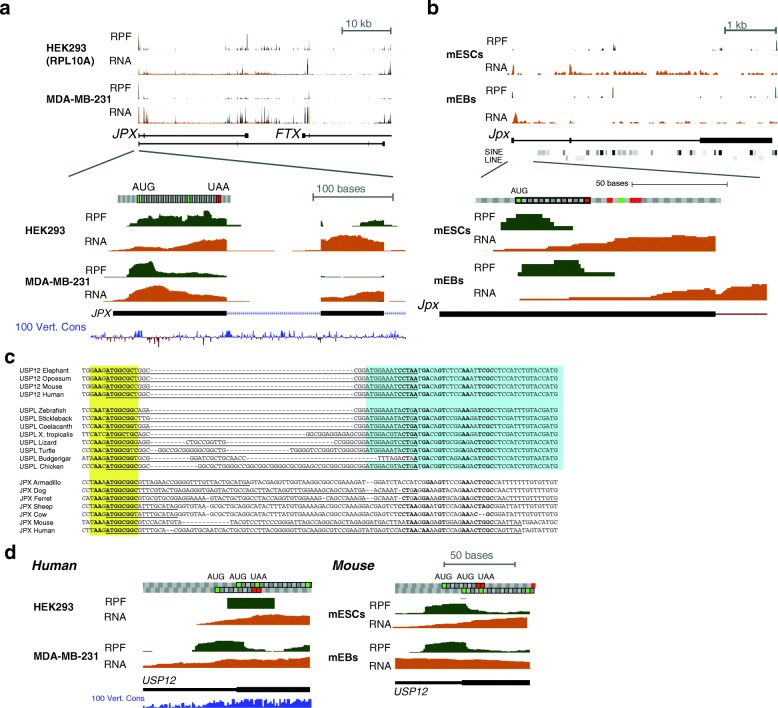
A conserved translated ORF at the 5′ end of the JPX lncRNA has evolved from an upstream ORF (uORF) in the USPL gene. **a** Ribosome protected fragment (*RPF*) and RNA-seq coverage across the JPX locus in human. Ribo-seq and RNA-seq data from HEK293 cells (RPL10A pulldown for Ribo-seq) [28] and MDA-MB-231 [29]. The codons in the ORF are color-coded above the tracks as in the UCSC genome browser: a green square indicates an AUG codon and a red box indicates a stop codon. **b** Same as in **a** for the mouse locus. Ribo-seq and RNA-seq data from mouse embryonic stem cells (*mESCs*) and mouse embryoid bodies (*mEBs*) [27]. **c** Sequence alignment between the uORFs in USP12 and USPL and the ORF in JPX lncRNAs from different species. Sequences were obtained from the UCSC whole genome alignments and from manual inspection of transcripts in syntenic regions, aligned using CLUSTALW, and the alignment was manually refined. The TISU element region is in yellow and the beginning of the annotated coding sequences of the USP genes is in blue. **d** Same as **a** and **b** but for the human and mouse USP12 5′ UTR

JPX is one of the lncRNAs in the X-inactivation center, derived from the USPL GLCP [19] and reported to positively regulate XIST expression, as the deletion of the JPX locus impairs X-inactivation [25, 26]. When examining ribosome footprinting data from a variety of human and mouse cell lines [27–29] and evaluating footprint distribution, we noted an efficiently translated ORF in the first exon of JPX (~20 nt downstream of the transcription start site), with limited evidence of translation downstream of it (Fig. 5a, b). The sequence surrounding the start codon of this ORF—T**AAG** **ATGGCGGC**G—matches 11 out of 12 bases of the translation initiator of short 5′ UTR (TISU) motif (S**AASATGGCGGC, S=G or C**) that is associated with both efficient translation initiation of ORFs in close proximity to the transcription start site and with transcriptional regulation by the YY1 transcription factor [30–32]. The regions around the ORF in the first exon are the only sequences similar between the human JPX and that of mouse and other mammals, as the majority of the human JPX is derived from primate-specific transposable elements. We found no evidence of the conservation of the peptide produced by the ORF among mammals, where the length of the ORF and the peptide sequence were both highly variable, suggesting that, akin to other translated ORFs in lncRNAs [33], the product of the translation is unlikely to be a stable and functional peptide.

The ORF-bearing region in the first exon of JPX has detectable sequence similarity with the first exon of the chicken USPL gene (Fig. 5c). As mentioned above, two clusters homologous to the LNX3/USPL/WAVE1 cluster are found throughout vertebrates. We therefore compared the sequences of the first exons of the USPL, its paralogs USP12 and USP46, and JPX lncRNAs throughout vertebrates. Interestingly, the JPX ORF aligned to a highly conserved upstream ORF (uORF) in the 5′ UTRs of USPL and USP12 genes, where the ORF typically overlapped in a different frame the major AUG start codon of USP genes. This uORF is also associated with a TISU element in the human and mouse USP12 (Fig. 5d; Additional file 1: Figure S6) and in human USP12 the uORF is more highly translated than the main ORF (Additional file 1: Figure S6), suggesting that this uORF may play a conserved role of regulating translation.

**Fig. 6:**
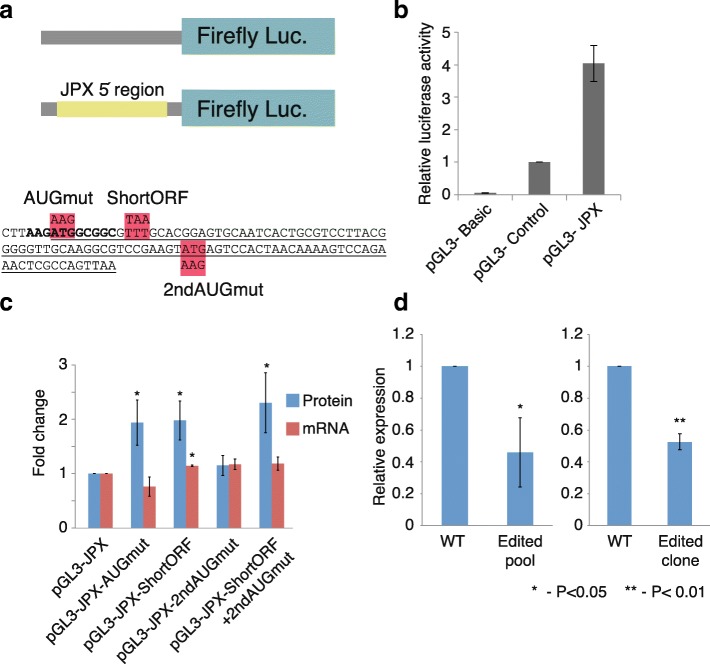
Translation repressive sequence elements are preserved in the 5′ end region of the JPX lncRNA. **a** Scheme and sequences of the JPX regions inserted into the pGL3 basic plasmid. On the bottom, the 5′ end of the insert with the mutated codons highlighted. The TISU element is in *bold* and the ORF is *underlined*. **b** Firefly luciferase levels (normalized by co-transfected Renilla luciferase and by the *pGL3-Control* levels). **c** Changes in firefly luciferase relative levels following the indicated mutations, normalized to co-transfected Renilla luciferase and to the pGL3-JPX construct; **d** Quantification of relative JPX transcript levels in HEK293 mutant cells compared to wild-type (*WT*) cells. β-Actin served as an internal control; bars show average ± SEM, n = 3

To test whether this translation-regulatory function is still present in the JPX lncRNA sequence, we cloned the promoter and the first two exons of JPX upstream of a firefly luciferase (see “Methods”; Fig. 6a) and mutated different parts of the ORF. Robust luciferase activity when the JPX 5′ end was placed in a promoterless vector confirmed that the proximal promoter of JPX is sufficient for driving transcription (Fig. 6b). Mutation of the AUG codon resulted in slightly decreased transcription of the luciferase mRNA and substantially increased luciferase activity, suggesting that the AUG within the TISU element contributes to suppression of translation downstream of the ORF (Fig. 6c). Mutation of the AUG to AAG in JPX in the endogenous context in HEK293 cells using CRISPR/Cas9 resulted in reduction in JPX expression levels, which was observed in a pool of edited cells and in individual clones (Fig. 6d; Additional file 1: Figure S7). The sequence JPX inherited from its USPL ancestor has thus retained the ability to drive transcription, be efficiently translated (as evident in the ribosome footprinting data), and to substantially repress translation downstream of the ORF (as evident both in the ribosome footprinting data of endogenous JPX and in the luciferase reporter assay). Further interrogation of the functional importance of the endogenous JPX will be required to elucidate the functional importance of this repression, but we expect that it may play an important role in preventing the translation machinery from affecting downstream elements and/or from triggering transcript degradation [33]. We thus propose that one type of sequences that some lncRNAs could have inherited from GLCPs are regulatory sequences that allow regulation of downstream translation. Notably, additional putative GLCP-derived human lncRNAs contain regions with experimental evidence of translation (taken from [34, 35]; Additional file 4: Table S4).

## Discussion

By systematically comparing conserved mammalian lncRNAs with protein-coding genes lost before and relatively soon after the emergence of the mammalian clade, we are now able to estimate how many conserved mammalian lncRNAs have evolved from remnants of protein-coding genes. Our simulations suggest that the recovery of syntenic pairs using our method is > 69% and hence the rate of false negatives (i.e., real GLCP-derived lncRNAs that are not called as syntenic) is relatively low. We thus can estimate that < 5% of the conserved mammalian lncRNAs are derived from loci that used to produce lost protein-coding genes. Some of those inherited features that may have contributed to their functionality, such as transcriptional regulatory elements and translation regulatory elements that may facilitate stability or functionality of the RNA. These and other features rarely require preservation of long stretches of DNA sequence, and so sequence similarity between lncRNAs and the descendants of their putative protein-coding ancestors is rare and weak. Other remnants of lost protein-coding genes are present in mammalian genomes in the form of annotated unprocessed pseudogenes, and these are typically excluded from lncRNA sets for various technical reasons, despite some of them also having important functions as noncoding RNAs [15, 36–39]. Unlike lncRNAs, unprocessed pseudogenes are not filtered based on their protein-coding potential, and there is increasing evidence that some of them are in fact still translated [35, 40, 41]. It is important to point out that we do not suggest that GLCP-derived lncRNAs and annotated unprocessed pseudogenes form two distinct groups, but rather present a continuum of coding sequence deterioration following the loss of the original full-length coding sequence.

Despite our integrated use of gene order and whole-genome alignments, it is important to note that our methodology is still limited by its signal-to-noise ratio, and the synteny-based method has an FDR of ~ 70%, and so a substantial fraction of the observed syntenic GLCP–lncRNA pairs are expected by chance (Fig. 2b, c). Increasing the specificity of detection of lncRNAs derived from GLCPs will require more sensitive methods for multiple genome comparisons (for better identification of disruptors) or better methods for detection of subtle sequence homology, potentially by pooling information from multiple species (such methods were recently used to look for homologs of individual lncRNAs [42], but are not available on a global scale). The differences in gene expression and other features between the GLCP-derived and other lncRNAs will likely become even more significant when such improved methods become available.

We note that for the sake of increased detection power, we focused here only on events of lncRNA derivation that were associated with loss of the ancestral gene. A related mechanism is duplication of a protein-coding gene followed by pseudogenization. This route is much more difficult to quantify. Better techniques for comparison of loci, e.g., ones that will be able to leverage information across multiple genomes, might increase the sensitivity of sequence similarity detection and shed further light on additional cases of derivation of lncRNAs from protein-coding loci.

**Fig. 7:**
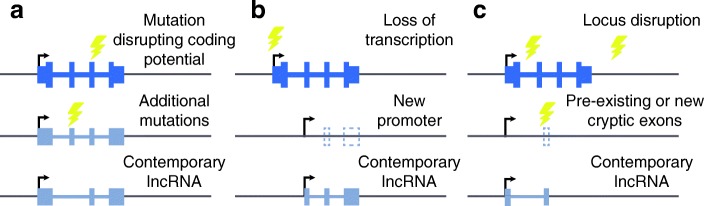
Possible modes of lncRNA emergence following coding potential loss. **a** A mutation could disrupt the functional protein production and be followed by additional mutations, further altering the sequence and the splicing pattern. **b** A genetic change can inactivate the promoter, which would lead to loss of mRNA production, and emergence of a new promoter, de novo or from a transposable element insertion, can yield a new gene that may re-use some of the remaining exons of the lost protein-coding gene. **c** A locus can be disrupted through genome rearrangements, juxtaposing the promoter of the lost protein-coding gene with new exonic sequences

An intriguing question that remains very difficult to answer is the sequence of events underlying the transition from a protein-coding gene into a lncRNA, as various scenarios are possible (Fig. 7). One possibility is a direct transition, where the acquisition of a lncRNA gave an initial selective advantage that helped drive the event to fixation. However, it is equally and perhaps even more plausible that loss of coding potential occurred first, perhaps with the aid of compensation offered by other homologs from the same family, and the lncRNA was acquired later, borrowing building blocks from the broken-down protein-coding gene, akin to a new house built from the stones of a shattered one. This second scenario is consistent with the generally common genesis of new lncRNAs during vertebrate evolution. The evolutionary construction of a lncRNA locus generally requires an active promoter (which can be adapted from a bidirectional promoter [43] or from an enhancer [44]), elements controlling splicing, and polyadenylation signals. Many of these elements are frequently adopted from transposable elements [3, 45–47], and since the sequence elements they require are short, they can also evolve from non-transcribed sequences. In this manner, GLCP-derived lncRNAs presumably contain a mixture of elements derived from the GLCP with elements adopted from other sources. The presence of sequence elements with other overlapping constraints, such as enhancers, can serve as an evolutionary “bridge” maintaining some of the sequence elements in the period between loss of protein-coding potential and acquisition of a lncRNA. We note that lncRNAs acquired this way are not necessarily functional. A third and particularly intriguing yet probably rare scenario is that the noncoding functionality was present before the loss of the protein-coding potential, and the gene was a bifunctional RNA [15, 48] prior to the loss. Examples that support such a scenario will not only make GLCP–lncRNA pairs of great significance from the lncRNA functionality point of view, but also shed light on GLCP functionality. However, the experimental testing of such cases at the time scales we consider here is made difficult by the limitations of experimental tools for carefully dissecting molecular functions in most of the reference species we have used in this study.

## Conclusions

We establish pseudogenization of genes that occurred over 100 million years ago as a non-negligible source of new lncRNAs that resulted in dozens of conserved and therefore possibly functionally important lncRNAs. In the future, the availability of thousands of additional vertebrate genome sequences, expected as part of the vertebrate Genome 10 K project [49], along with improved methodologies for describing and comparing short functional elements in long RNAs, will shed further light on the composition and origins of the vibrant milieu of mammalian lncRNAs.

## Methods

### Genome assemblies

All the analyses were done using the hg19, rheMac3, calJac3, mm9, oryCun2, canFam3, murFur1, ornAna3, monDam5, galGal4, anoCar2, xenTro3, latCha1, danRer7, gasAcu1, oryLat2, and oreNil2 assemblies, and where annotations were available in other assemblies, they were mapped to those assemblies using the liftOver tool from the UCSC tools. Pairwise genome alignments were obtained from the UCSC genome browser. For identifying syntenic gene pairs, mouse mm9 coordinates were mapped to mm10 coordinates and pairwise alignments with mm10 were used.

### Identification of lost protein-coding genes

Homologies among protein-coding genes were obtained from Ensembl Compara version 80. We defined eight core mammalian species (human, rhesus, marmoset, mouse, rabbit, dog, sheep and ferret), and four core fish species (zebrafish, stickleback, tilapia, and medaka). Criteria for presence/absence of the genes in mammals and other vertebrates are shown in Additional file 1: Figure S1a. For all the species except dog, we allowed presence in one mammalian genome, and allowed for omissions in some of the vertebrate species to accomodate some annotation errors. In practice, in most cases, GLCPs had no annotated homologs in any mammalian genomes currently present in Ensembl Compara (Additional file 1: Figure S1b).

### lncRNA and pseudogene set

In order to focus on relatively reliable lncRNA annotations, we used lncRNAs that we previously annotated using PLAR [3], excluding transcripts antisense to protein-coding genes. In order to focus on conserved lncRNAs, we only considered 2740 and 1163 human and mouse lncRNAs, respectively, that were conserved by sequence in other species (similar in sequence to lncRNAs from other species rather than simply having alignable DNA sequence in the other species), after excluding human lncRNAs conserved only in primates, and after excluding lncRNAs where the fraction of the sequence covering tandem repeats (taken from the “Simple Repeats” track in the UCSC genome browser) exceeded 50%. We also extracted all transcripts annotated as “unprocessed_pseudogene” (including those annotated as transcribed) from Ensembl version 82 in human and mouse. These were further filtered and single exon transcripts shorter than 500 nucleotides were removed.

### Sequence comparisons

Sequence comparisons were performed using three tools: BLASTN, TBLASTX (from the BLAST 2.2.28+ package), and SSEARCH (version 36.3.7b). BLASTN was used with the parameters “-word_size 8 -strand plus -evalue 1e-5” and hits with E-value below 10^−5^ were considered significant. TBLASTX was used with the parameters “-word_size 2 -strand plus” and hits with E-value below 10^−5^ were considered significant. SSEARCH36 was used with the default parameters and hits with E-value below 10^−10^ were considered significant. In order to evaluate the false discovery rate, for each lncRNA-GLCP or pseudogene-GLCP pair, we selected ten random GLCPs and aligned the sequences using the same parameters. We then computed the empirical false discovery rate (Additional file 1: Figure S4b) as the ratio between the average number of random pairs and the pairs in the real dataset that had sequence similarity below each threshold.

### Gene expression estimations

Expression levels of Ensembl genes were computed using RSEM [50] applied to RNA-seq datasets described in the “Availability of data and materials” section. Gene-level gene expression estimates were used for expression level quantification. Pseudogenes were defined as expressed if they had an RPKM of at least 1.0 in at least one human/mouse tissue.

The tissue specificity index of GLCPs and lncRNAs was calculated as previously described [51], based on expression levels in at least five different tissues in each species.

### Identification of syntenic gene pairs

Homologous gene pairs were obtained from Ensembl Compara 80. For a query gene (i.e., a GLCP) *G*_*Q*_ and a potential target gene *G*_*T*_ (e.g., a conserved lncRNA or an unprocessed pseudogene), we first identified among the protein-coding genes conserved in the other species those that overlap *G*_*Q*_ and *G*_*T*_ and the closest non-overlapping protein-coding neighbors on either side (Additional file 1: Figure S3a). We denote those groups *Overlapping*(*G*_*X*_), *Upstream*(*G*_*X*_), and *Downstream*(*G*_*X*_). The distance to the closest neighboring gene was restricted to 500 kb in human and in each of the other species it was scaled by the ratio between the size of the genome of the species and the size of the human genome. Further, we did not allow neighbors across unbridged gaps in the genome assembly. This was done to accommodate genomes like the opossum genome where unrelated contigs are all placed in an “chrUn” chromosome separated by unbridged gaps.

We considered *G*_*Q*_ and *G*_*T*_ *potentially syntenic* if there were common genes with the same relative orientation to *G*_*Q*_ and to *G*_*T*_ between: (i) the homologs of *Upstream*(*G*_*Q*_)∨*Overlapping*(*G*_*Q*_) and *Upstream*(*G*_*T*_)∨*Overlapping*(*G*_*T*_); or (ii) the homologs of *Downstream*(*G*_*Q*_)∨*Overlapping*(*G*_*Q*_) and *Downstream*(*G*_*T*_)∨*Overlapping*(*G*_*T*_)*.* Query-target pairs with at least one matching gene were carried forward.

For each potential syntenic query–anchor pair, we used chains of pairwise alignments between the genome sequences of the two species. We first pre-processed these chains, and split them in any position where the gap in one of the species was larger than 10 kb. We then looked for “disruptors”—chains that appear in contradictory orientations when considering the region spanning between the query/target gene and the adjacent protein-coding genes (Fig. 2a; Additional file 1: Figure S3b). Specifically, we obtained chains of pairwise alignments from the UCSC genome browser. For each *G*_*X*_ we identified all the chains in the genomic interval spanning from *Upstream*(*G*_*X*_) to *Downstream*(*G*_*X*_), including an additional 100 kb on each side, and divided them into those that (i) overlap, (ii) are downstream of *G*_*X*_, and (iii) are upstream of *G*_*X*_, with further divisions based on the strand on which the aligned sequence appeared. Disruptor chains were those chains that aligned regions with the same relative orientation (Additional file 1: Figure S3b): (i) upstream of *G*_*Q*_ and downstream of *G*_*T*_; (ii) downstream of *G*_*Q*_ and upstream of *G*_*T*_; (iii) upstream or downstream of *G*_*Q*_ and overlapping *G*_*T*_; (iv) overlapping *G*_*Q*_ but upstream or downstream of *G*_*T*_. Any pair of potentially syntenic genes that had at least one disruptor were not considered further.

### Randomization and FDR estimation

In order to measure the number of syntenic pairs expected by chance, we compared the number of syntenic GLCP–lncRNA and GLCP–pseudogene pairs to the numbers obtained when using a set of randomly placed lncRNAs or pseudogenes and/or a set of randomly selected genes instead of the GLCPs.

Random placement of lncRNAs/pseudogenes was performed using the same method we used previously [52]—on each chromosome, we first clustered overlapping transcript models (isoforms) into “bundles” and then iteratively placed each bundle in a random location on the same chromosome so that it would not overlap annotated lncRNAs, protein-coding genes, or already placed bundles.

Random sets of protein-coding genes were obtained by randomly selecting the same number of protein-coding Ensembl genes (ENSG identifiers) as the number of GLCPs.

### Cell culture and transfections

HEK293 cells were cultured in DMEM (Gibco) supplemented with 10% fetal bovine serum (Gibco) and 1% Pen-Strep (Gibco) and passaged 1:8 every 3–4 days. Transfections were performed using PolyEthylene Imine 3 (PEI linear, Mr 25000 from Polyscience Inc).

### Cloning

The wild-type promoter up to the second exon of the human gene JPX was cloned into pGL3-basic (Promega catalog number E1751) upstream of the luciferase gene using the XhoI and HindIII restriction sites. Specifically designed primers (Additional file 1: Table S5) were used to amplify the desired areas from human genomic DNA (chrX:73,943,696–73,944,644 in the hg19 assembly). The cut amplicon was purified with QIAquick PCR Purification Kit (QIAGEN). The cut plasmid was treated with CIP (NEB) and purified using QIAquick Gel Extraction Kit (QIAGEN). The cut and purified DNA fragments were ligated using Quick Ligation Kit (NEB) and transformed to competent bacteria (NEB). Point mutations were conducted using the QuikChange Lightning Site-Directed Mutagenesis Kit (Agilent Technologies) according to the manufacturer’s protocol, with primer (Sigma Aldrich) designed with QuikChange Primer Design (http://www.genomics.agilent.com/primerDesignProgram.jsp).

### Luciferase assays

For luciferase assays, HEK293 cells were plated in 24-well plates (200,000 per well), and for RNA extraction in 12-well plates (400,000 per well). After 24 h each individual pGL3 plasmid was co-transfected with pIS1 (Addgene plasmid number 12179) as internal control. The plasmids used were pGL3-basic (Promega catalog number E1751), pGL3-control (Promega catalog number E1741) and the JPX pGL3 constructs described in the previous section. Luciferase activity was recorded 24 h post-transfection using the Dual-Glo luciferase Assay System (Promega) in the Microplate Luminometer device (Veritas). Firefly luciferase signal/Renilla luciferase signal was calculated for each sample. Fold change is relative to the wild-type JPX construct.

### Real-time PCR analysis of gene expression

Total RNA was isolated using TRI reagent (MRC) or with the RNeasy Mini Kit (QIAGEN), and then reverse transcribed using random primers (Quanta), according to the manufacturer’s protocols. Real-time PCR was conducted using Fast SYBR qPCR mix (Life Technologies). The primer sets that were used are listed in Additional file 1: Table S5. The assays contained 10 ng sample cDNA in a final volume of 10 μl and were run on AB qRT-PCR system ViiA 7 (Applied Biosystems). Relative expression levels were normalized to Renilla levels.

### Genome editing of the endogenous JPX ORF

The CRISPR/Cas9 system was used to mutate the first ATG codon in the JPX ORF in HEK293 cells. Construction of single guide RNA (sgRNA) plasmid was done following the Zhang Lab General Protocol (https://media.addgene.org/cms/filer_public/e6/5a/e65a9ef8-c8ac-4f88-98da-3b7d7960394c/zhang-lab-general-cloning-protocol.pdf) using pLKO.1-puro U6 sgRNA CAG (Addgene number 50927). gRNA sequence was designed to complement targeted sequence with least off-targets possible using CHOPCHOP^63^ (https://chopchop.rc.fas.harvard.edu/).

sgRNA (200 ng) was co-transfected with Cas9-Puro (2 μg; Addgene number 62988) and with the following oligo: CACCCCCGGCTTTCATCCGCCTATGCCCTAGGGCTAGTGGAAGACTTAAGAaccCGGCGTTTGCACGGAGTGCAATCACTGCGTCCTTACGGGGGTTGCAAGG.

One day after the transfection Puromycin selection was applied to the cells at a concentration of 1 μg/ml for 3 days and the surviving cells were transfected again. After 6 additional days of selection, the cells were harvested for genotyping or RNA extraction for RT-PCR. Genotyping was performed using the BccI restriction enzyme (NEB) that recognizes the GATGG sequence that is present only in the wild-type alleles but not in the mutated edited alleles (Additional file 1: Figure S7). We also isolated single clones, genotyped them, and tested JPX expression using RT-PCR.

## Additional files

Additional file 1: Supplementary Figures and Tables. **Figure S1.** GLCP search scheme. **Figure S2.** Comparison of maximum expression levels of protein-coding gene groups. **Figure S3.** Identifying and filtering syntenic pairs using whole-genome alignments. **Figure S4.** Effect of sequence similarity threshold on the number of sequence pairs in real and permuted data that have significant similarity. **Figure S5.** Correlation between the expression levels of human GLCPs and putative GLCP-derived lncRNAs. **Figure S6.** 5′ ends of ribosome footprints at the first two exons of Ups12 mRNA. **Figure S7.** Genotyping of HEK293 cells with mutations in the JPX ORF. **Table S3.** Fraction of transcripts with support of their 5′ ends. **Table S5.** Primer sequences. (DOCX 6795 kb)

Additional file 2: **Table S1.** GLCPs in each of the six reference species. (XLSX 104 kb)

Additional file 3: **Table S2.** Coordinates of the conserved lncRNAs from human and mouse considered in this study (in BED format). (XLSX 1079 kb)

Additional file 4: **Table S4.** Positionally conserved GLCP–lncRNA and GLCP–pseudogene pairs. (XLSX 188 kb)

## Abbreviations

FDR: False discovery rate
GLCP: Gene with lost coding potential
lncRNA: Long non-coding RNA
PLAR: Pipeline for identification of lncRNAs from RNA-seq data
RPKM: Reads per kilobase per million mapped reads
sgRNA: Single guide RNA
TISU: Translation initiator of short 5′ UTR
uORF: Upstream ORF
XIC: X inactivation center.
