# Supplementary material for: A subset of conserved mammalian long non-coding RNAs are fossils of ancestral protein-coding genes
Source: Genome Biol. 2017 Aug 30;18:162. doi: 10.1186/s13059-017-1293-0 (PMC5577775; doi:10.1186/s13059-017-1293-0)
Supplement: Supplementary file 1 — Supplementary Figures and Tables. Figure S1. GLCP search scheme. Figure S2. Comparison of maximum expression levels of protein-coding gene groups. Figure S3. Identifying and filtering syntenic pairs using whole-genome alignments. Figure S4. Effect of sequence similarity threshold on the number of sequence pairs in real and permuted data that have significant similarity. Figure S5. Correlation between the expression levels of human GLCPs and putative GLCP-derived lncRNAs. Figure S6. 5′ ends of ribosome footprints at the first two exons of Ups12 mRNA. Figure S7. Genotyping of HEK293 cells with mutations in the JPX ORF. Table S3. Fraction of transcripts with support of their 5′ ends. Table S5. Primer sequences. (DOCX 6795 kb) [file 13059_2017_1293_MOESM1_ESM.docx]

**Supplementary information for “A subset of conserved mammalian long non-coding RNAs are fossils of ancestral protein-coding genes” by Hezroni et al.**

**Figure S1. GLCP search scheme. (A)** The species in which the genes were required to be found (in green) and not found (in red), with the numbers indicating the number of species where the gene had to be present/lost in. **(B)** Distributions of the fractions of all species annotated in each group in Ensembl Compara in which homologs of the genes are present.

**Figure S2**. **Comparison of maximum expression levels of protein-coding gene groups.** As in Figure 1C, comparing maximum expression levels of the indicated protein-coding genes in the reference species. P-values computed using Wilcoxon rank-sum test. All comparisons indicated with asterisks are significant at FDR<0.05 (Benjamini-Hochberg).

**Figure S3. Identifying and filtering syntenic pairs using whole-genome alignments. (A)** The scheme used for identifying syntenic GLCP-lncRNA pairs. **(B)** The different types of “disrupting” pairwise genome alignment chains, which are found in an inconsistent location relative to the GLCP and the lncRNA, or overlap one of the genes in the pair, but not the other. **(C)** Total numbers of syntenic GLCP-lncRNA and GLCP-unprocessed pseudogene pairs (those that passed the first phase) in human (top) and mouse (bottom) and the numbers of pairs that passed the second disruptor-based filtering test.

**Figure S4. Effect of sequence similarity threshold on the number of sequence pairs in real and permuted data that have significant similarity. (A)** Numbers of GLCP-lncRNA or GLCP-Pseudogene pairs (summed over all six species) passing the indicated threshold when using the SSEARCH, BLASTN or TBLASTX algorithms. The selected threshold is indicated in bold. For BLASTN and GLCP-pairs, no pairs were significant in the permuted data. **(B)** Same as A, showing the ratio between the average number of significant pairs in the permuted and the total number of significant pairs in real data.

**Figure S5.** **Correlation between the expression levels of human GLCPs and putative GLCP-derived lncRNAs.** In reference species where over 15 pairs of GLCPs and GLCP-derived lncRNAs were identified (lizard, chicken, coelacanth and *X. tropicalis*), Spearman’s correlation coefficient was computed between expression levels of the lncRNAs in human tissues and the GLCPs in the reference species tissues for all tissues for which datasets were available in both of the species. See Methods for details about the RNA-seq datasets used in each species.

**Figure S6. 5' ends of ribosome footprints at the first two exons of Ups12 mRNA.** Ribosome protected fragments from HEK293 cells ([Lee et al. 2012](#_ENREF_4)) and HCT116 cells ([Koch et al. 2014](#_ENREF_3)) obtained after treatment with lactimidomycin (LTM) that enriches for footprints at the first translated codon and after treatment with cyclohexamide (CYH).


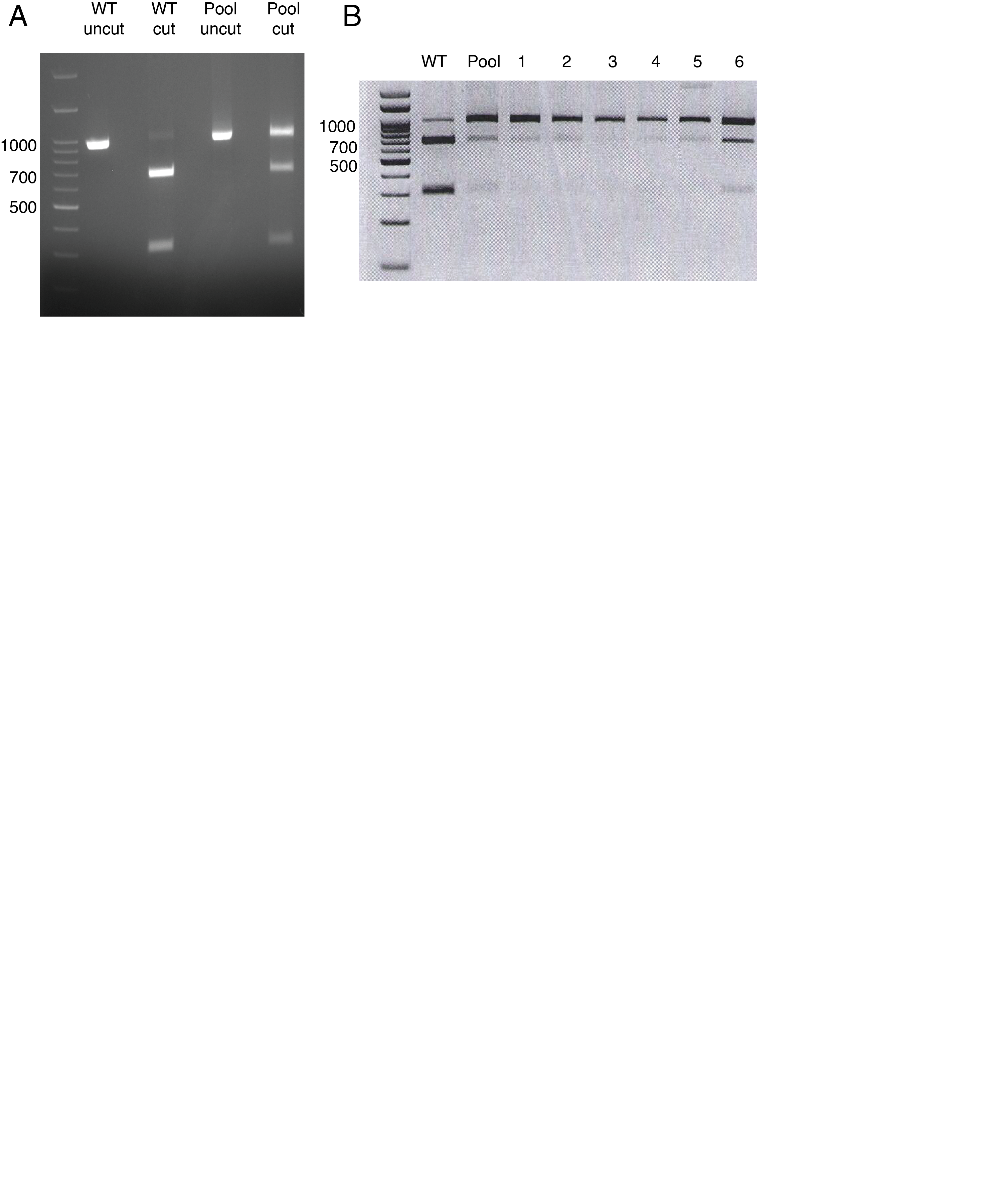


**Figure S7. Genotyping of HEK293 cells with mutations in the JPX ORF. (A)** PCR productions before (uncut) and after (cut) incubation with the BccI restriction enzyme recognizing the GATGG sequence. **(B)** PCR products of WT, pool and individual clones following incubation with BccI. Clone number 2 was used in RT-PCR experiments.

**Table S3. Fraction of transcripts with support of their 5' ends.** Support evaluated using CAGE data from the FANTOM5 project ([Consortium et al. 2014](#_ENREF_1)) (phases 1+2) and 3' end data from 3P-seq ([Nam et al. 2014](#_ENREF_5)) (human) or PolyA-seq ([Derti et al. 2012](#_ENREF_2)) (human and mouse) read clusters. A transcript end was considered as experimentally supported if a CAGE/3P-seq read cluster was mapped on the correct strand within 500 nt of the transcript model.

|  | **Human conserved lncRNAs** | **All human lncRNAs** | **Mouse conserved lncRNA** | **All mouse lncRNAs** |
| --- | --- | --- | --- | --- |
| **5' support** | 53.9% | 35.2% | 58.8% | 40.8% |
| **3' support** | 65.3% | 48.0% | 72.8% | 62.0% |
| **Both 5' and 3' support** | 44.1% | 24.9% | 51.5% | 37.8% |

**Table S5**. **Primer sequences**

| qPCR | PIS1 spliced Renilla F | ACCACTGCGGACCAGTTATCATCC |
| --- | --- | --- |
|  | PIS1 spliced Renilla R | ggcactgggcaggtgtccac |
|  | Spliced Firefly in JPX constructs F | TCGCCAGTTAATAGTATTGTGTCTC |
|  | Spliced Firefly in JPX constructs R | AACAGTACCGGAATGCCAAG |
| Cloning | JPX first exons F | aaaactcgagCATTGACGGACTCACTGTATG |
|  | JPX first exons R | accaaaagcttTTCGTCAGTAGAAGTTAGGCG |
| Mutagenesis | JPX_AUGmut F | GGGCTAGTGGAAGACTTAAGAAGGCGGCGTTTG |
|  | JPX_AUGmut R | CAAACGCCGCCTTCTTAAGTCTTCCACTAGCCC |
|  | JPX_EarlyStop F | GACTTAAGATGGCGGCGTAAGCACGGAGTGCAATCA |
|  | JPX_EarlyStop R | TGATTGCACTCCGTGCTTACGCCGCCATCTTAAGTC |
|  | JPX_2ndAUGmut F | TTTGTTAGTGGACTCTTACTTCGGACGCCTTGCAA |
|  | JPX_2ndAUGmut R | TTGCAAGGCGTCCGAAGTAAGAGTCCACTAACAAA |
| Endogenous JPX qPCR | JPX_qPCR_F | TAATAGTATTGTGTCTCTTCAAAATATC |
|  | JPX_qPCR_R | CTTCTGCAACTTCCAAGCTTC |
|  | Actin_F | CCCTGGACTTCGAGCAAGAG |
|  | Actin_R | ACTCCATGCCCAGGAAGGAA |
| JPX Genotyping | JPX_Genotype_F | AAAACTCGAGCATTGACGGACTCACTGTAT G |
|  | JPX_Genotype_R | CCAAGCTTCGTCAGTAGAAGT |

**References**

Consortium F, the RP, Clst, Forrest AR, Kawaji H, Rehli M, Baillie JK, de Hoon MJ, Lassmann T, Itoh M et al. 2014. A promoter-level mammalian expression atlas. *Nature* **507**(7493): 462-470.

Derti A, Garrett-Engele P, Macisaac KD, Stevens RC, Sriram S, Chen R, Rohl CA, Johnson JM, Babak T. 2012. A quantitative atlas of polyadenylation in five mammals. *Genome research* **22**(6): 1173-1183.

Koch A, Gawron D, Steyaert S, Ndah E, Crappe J, De Keulenaer S, De Meester E, Ma M, Shen B, Gevaert K et al. 2014. A proteogenomics approach integrating proteomics and ribosome profiling increases the efficiency of protein identification and enables the discovery of alternative translation start sites. *Proteomics* **14**(23-24): 2688-2698.

Lee S, Liu B, Lee S, Huang SX, Shen B, Qian SB. 2012. Global mapping of translation initiation sites in mammalian cells at single-nucleotide resolution. *Proceedings of the National Academy of Sciences of the United States of America* **109**(37): E2424-2432.

Nam JW, Rissland OS, Koppstein D, Abreu-Goodger C, Jan CH, Agarwal V, Yildirim MA, Rodriguez A, Bartel DP. 2014. Global analyses of the effect of different cellular contexts on microRNA targeting. *Molecular cell* **53**(6): 1031-1043.
